# Our Editorial Staff

**Published:** 1874-01

**Authors:** 


*Our Editorial Staff.*

With this issue of the RECORD a new feature in its editorial management has been inaugurated. It will be seen that a number of medical gentlemen whose reputations for honesty, intelligence and learning are well known, have consented to represent their States and sections as editors of our journal. In doing this, there is no conflict between them and our Associate and Corresponding Editors. There is intended by the arragement, an increase of work upon the basis of equality and harmony. The editors have agreed to contribute *regularly* for its columns, while our other editorial friends have only agreed to contribute at their convenience. All will represent and look after the interests of the RECORD in their localities, and aid in giving influence, reputation and circulation to a journal which we earnestly wish and desire them to feel belongs to them. The RECORD is not a machine for money-making. It is not run as a prop to sustain any ring or clique, nor to boalster up the tottering fortunes of any pet School of Medicine. It is independent and free; praising where praise is due, and outspoken in its condemnation of those principles (not men) which if not arrested are certain to destroy the unity of the profession at the shrine of selfishness.

We ask cordially, earnestly, respectfully, that our brethren will give us and our journal their hearty support and co-operation. They can give it an impetus which will carry it, in point of influence and circulation, beyond any other journal on the continent.

We ask our Editors and Associates as well as our host of readers to form the line and let the word go from lip to lip and heart to heart—"Advance!"

